# Sympathetic innervation of the supraclavicular brown adipose tissue: A detailed anatomical study

**DOI:** 10.1371/journal.pone.0290455

**Published:** 2023-10-04

**Authors:** Shumpei Mori, Ryan S. Beyer, Breno Bernardes de Souza, Julie M. Sorg, Donald B. Hoover, Harold S. Sacks, Michael C. Fishbein, Grace Chang, Warwick J. Peacock, Maie A. St. John, James Law, Micheal E. Symonds, Olujimi A. Ajijola, Kalyanam Shivkumar, Preethi Srikanthan

**Affiliations:** 1 David Geffen School of Medicine at UCLA, UCLA Health System, University of California Los Angeles (UCLA) Cardiac Arrhythmia Center, Los Angeles, CA, United States of America; 2 Department of Biomedical Sciences, Quillen College of Medicine, East Tennessee State University, Johnson City, TN, United States of America; 3 Center of Excellence in Inflammation, Infectious Disease and Immunity, East Tennessee State University, Johnson City, TN, United States of America; 4 VA Endocrinology and Diabetes Division, Department of Medicine, UCLA, Los Angeles, CA, United States of America; 5 Department of Pathology and Laboratory Medicine, UCLA, Los Angeles, CA, United States of America; 6 Department of Surgery, UCLA, Los Angeles, CA, United States of America; 7 Department of Head and Neck Surgery, UCLA, Los Angeles, CA, United States of America; 8 Academic Unit of Population and Lifespan Sciences, Centre for Perinatal Research, School of Medicine, University of Nottingham, Nottingham, United Kingdom; 9 Nottingham Children’s Hospital, Nottingham University Hospitals NHS Trust, Nottingham, United Kingdom; 10 Division of Endocrinology UCLA, UCLA Health System, David Geffen School of Medicine at UCLA, Los Angeles, CA, United States of America; AIIMS: All India Institute of Medical Sciences, INDIA

## Abstract

**Background:**

The supraclavicular fossa is the dominant location for human brown adipose tissue (BAT). Activation of BAT promotes non-shivering thermogenesis by utilization of glucose and free fatty acids and has been the focus of pharmacological and non-pharmacological approaches for modulation in order to improve body weight and glucose homeostasis. Sympathetic neural control of supraclavicular BAT has received much attention, but its innervation has not been extensively investigated in humans.

**Methods:**

Dissection of the cervical region in human cadavers was performed to find the distribution of sympathetic nerve branches to supraclavicular fat pad. Furthermore, proximal segments of the 4^th^ cervical nerve were evaluated histologically to assess its sympathetic components.

**Results:**

Nerve branches terminating in supraclavicular fat pad were identified in all dissections, including those from the 3^rd^ and 4^th^ cervical nerves and from the cervical sympathetic plexus. Histology of the proximal segments of the 4^th^ cervical nerves confirmed tyrosine hydroxylase positive thin nerve fibers in all fascicles with either a scattered or clustered distribution pattern. The scattered pattern was more predominant than the clustered pattern (80% vs. 20%) across cadavers. These sympathetic nerve fibers occupied only 2.48% of the nerve cross sectional area on average.

**Conclusions:**

Human sympathetic nerves use multiple pathways to innervate the supraclavicular fat pad. The present finding serves as a framework for future clinical approaches to activate human BAT in the supraclavicular region.

## Introduction

Supraclavicular fat is the metabolically important and one dominant depot of human brown adipose tissue (BAT) [1, 2]. Activation BAT promotes non-shivering thermogenesis by expenditure of glucose and free fatty acid. Activation of BAT also induces secretion of so-called batokines which commonly target the liver, heart and skeletal muscle to upregulate basal metabolic rate [3]. In fact, BAT is increased in humans with cancers and heart failure [4]. This neurohormonal interaction may explain why activation of even a small amount of BAT can modulate metabolism in the whole body [3]. The discovery of human BAT at autopsy in 1972 [1] has evoked interest in regulation of its activation as a potential therapeutic target for metabolic disorders [5]. Activation of BAT is mediated by the sympathetic nervous system, which is well established in rodents. In rodents, regions including the lateral hypothalamus and the paraventricular nucleus play an important role in the central control of sympathetic innervation [6–11]. Signals from these nuclei are distributed by the hypothalamo-spinal tract to the medullary raphe and the intermediolateral nucleus of the spinal cord. Via the paraspinal sympathetic chain, preganglionic sympathetic fibers reach the stellate ganglion and branches of intercostal nerves before terminating in the interscapular BAT. However, neural and hormonal regulation of specific BAT depots in humans is not well established. This is, in part, because BAT depots in humans are not identical to those in mice, in terms of distribution, molecular and anatomic morphology, and innervation [12]. Functional magnetic resonance imaging studies in humans, under a whole-body cooling paradigm, suggest complicated hierarchical, thermoregulatory control systems involving the right anterior insula and red nucleus/substantia nigra region. These central regions normally exert tonic inhibition on the medullary raphe [13]. From this area, BAT is likely to receive innervation via the intermediolateral nucleus at the lateral horn of the spine and sympathetic ganglia and trunks in the thoracic and cervical region. To facilitate development of efficient methods to activate supraclavicular BAT, it is crucial to understand innervation pathways, in addition to establishing reliable diagnostic methodologies to evaluate BAT distribution and activity [14–18]. To the best of our knowledge, however, even the terminal portions of this innervation have yet to be fully investigated. Only a recent single study by Sievers et al. [19], demonstrated specific nerve branches from the cervical plexus terminating in supraclavicular fat pad. The purpose of our study was to extend these findings by defining the route sympathetic fibers take to innervate supraclavicular BAT and address their possible clinical significance.

## Methods

Three embalmed cadavers and five fresh cadavers were included in these dissections and histological studies (represented respectively in Tables 1 and 2). This observational study was approved by the institutional review board (IRB#20-000432). All data of donated cadavers were fully anonymized and requirement for informed consent was waived by the institutional review board.

**Table 1:** Dissection findings.

Case	Age (years)	Sex	Body height (cm)	Body weight (kg)	Body mass index (kg/m^2^)	Side	Origin of the branches innervating the supraclavicular fat
1	96	Female	155	50	20.8	Right	C3, C4, Cervical sympathetic plexus
						Left	C3, C4
2	93	Female	152	52	22.8	Right	C3, C4, Cervical sympathetic plexus
						Left	C3, C4
3	92	Female	173	50	16.7	Right	C3, C4, Cervical sympathetic plexus
						Left	C3, C4, Cervical sympathetic plexus

C3, 3rd cervical nerve; C4, 4th cervical nerve.

**Table 2:** Histological evaluation findings.

Case	Age (years)	Sex	Body height (cm)	Body weight (kg)	Body mass index (kg/m^2^)	Side	Major diameter of the nerve (mm)	Minor diameter of the nerve (mm)	Number of fascicles in the proximal segment of C4	clusterring	scattering	TH (+) area percentage
1	76	Male	178	54	17.0	Right	3.5	1.6	10	2	8	3.49%
						Left	3.8	2.3	8	0	8	8.08%
2	84	Male	178	73	23.0	Right	5.0	2.9	16	5	11	4.36%
						Left	4.7	4.0	6	2	4	1.70%
3	95	Female	165	54	19.8	Right	4.9	2.3	5	0	5	2.69%
						Left	6.0	3.7	21	1	20	1.72%
4	99	Female	155	32	13.3	Right	4.2	2.7	7	0	7	0.47%
						Left	4.2	2.9	11	5	6	1.43%
5	79	Male	178	64	20.2	Right	4.1	3.4	9	0	9	0.18%
						Left	2.8	1.2	3	0	3	0.72%
Average	87		171	55	18.7		4.3	2.7	10	2	8	2.48%

C4, 4th cervical nerve; TH, Tyrosine-Hydroxylase.

### Dissection of cervical region

Six neck dissections of the embalmed cadavers were performed to identify nerve branches distributed to supraclavicular fat pad. Dissection was performed with great attention to detail, with or without a surgical loupe of 2.5 times magnification. Briefly, systematic progressive layered dissection was performed in order of: (1) skin incision along the base of the mandible, median line of the neck, and clavicle, (2) dissection of the cutaneous nerves including the supraclavicular nerves and the cervical ansa, (3) dissection and removal of the sternocleidomastoid muscle, (4) dissection of the carotid artery and vagus nerve by opening the carotid sheath, (5) identification of the cervical sympathetic plexus by dissection of the fascia of the longus capitis/colli muscles, (6) identification of the phrenic nerve on the anterior scalene muscle, (7) dissection of the proximal part of the 4^th^ cervical nerve, followed by the 3^rd^ and 2^nd^ cervical nerves along the longus capitis/colli muscles, (8) dissection of the proximal brachial plexus (5^th^ to 8^th^ cervical nerves) between the anterior and middle scalene muscles, (9) dissection of the subclavian artery and its proximal branches, including the suprascapular artery, thyrocervical trunk, costocervical trunk, vertebral artery, and internal thoracic artery, and (10) dissection of the inferior cervical ganglion (superior half of the stellate ganglion) by removing the vertebral artery. During the procedure, the supraclavicular fat pad initially covered by the sternocleidomastoid muscle, trapezius muscle, and clavicle, was maintained as much as possible even though the dissection of nerves required removal of the surrounding fibroadipose tissue. During the dissection, the veins, lymphatics, and middle part of the clavicle were removed, if necessary, for better visualization of the supraclavicular fat pad and the nerve branches terminating within it. Histological evaluation to confirm identity of nerves was performed as necessary.

### Histological evaluation of the proximal 4^th^ cervical nerve

The dissection study revealed the 4^th^ cervical nerve consistently provided nerve branches to the supraclavicular fat pad. Ten proximal segments of the 4^th^ cervical nerves (10-mm in length) were additionally collected from five fresh cadavers. In order to accurately perform immunohistochemical staining, fresh cadavers were selected as immunohistochemical staining results are not reliable in long-term embalmed cadavers. The fresh nerve samples were then fixed in 10% formalin for 24-hour followed by preservation in 70% ethanol. The samples were aligned in the histology cassette to provide their cross-sectional images with hematoxylin & eosin and tyrosine hydroxylase (mouse monoclonal anti-tyrosine hydroxylase antibody, T1299, Sigma, St. Louis, MO, USA) stains. Digitally scanned images were then evaluated and quantitatively analyzed using commercially available software (Aperio ImageScope^TM^, Leica Biosystems, Deer Park, IL, USA).

### Statistical analysis

Due to the limited sample size, we refrained from applying extensive statistical evaluation. Rather, we simply provided the mean data without standard deviations for histological evaluation.

## Results

### Dissection of cervical region

**Fig 1:**
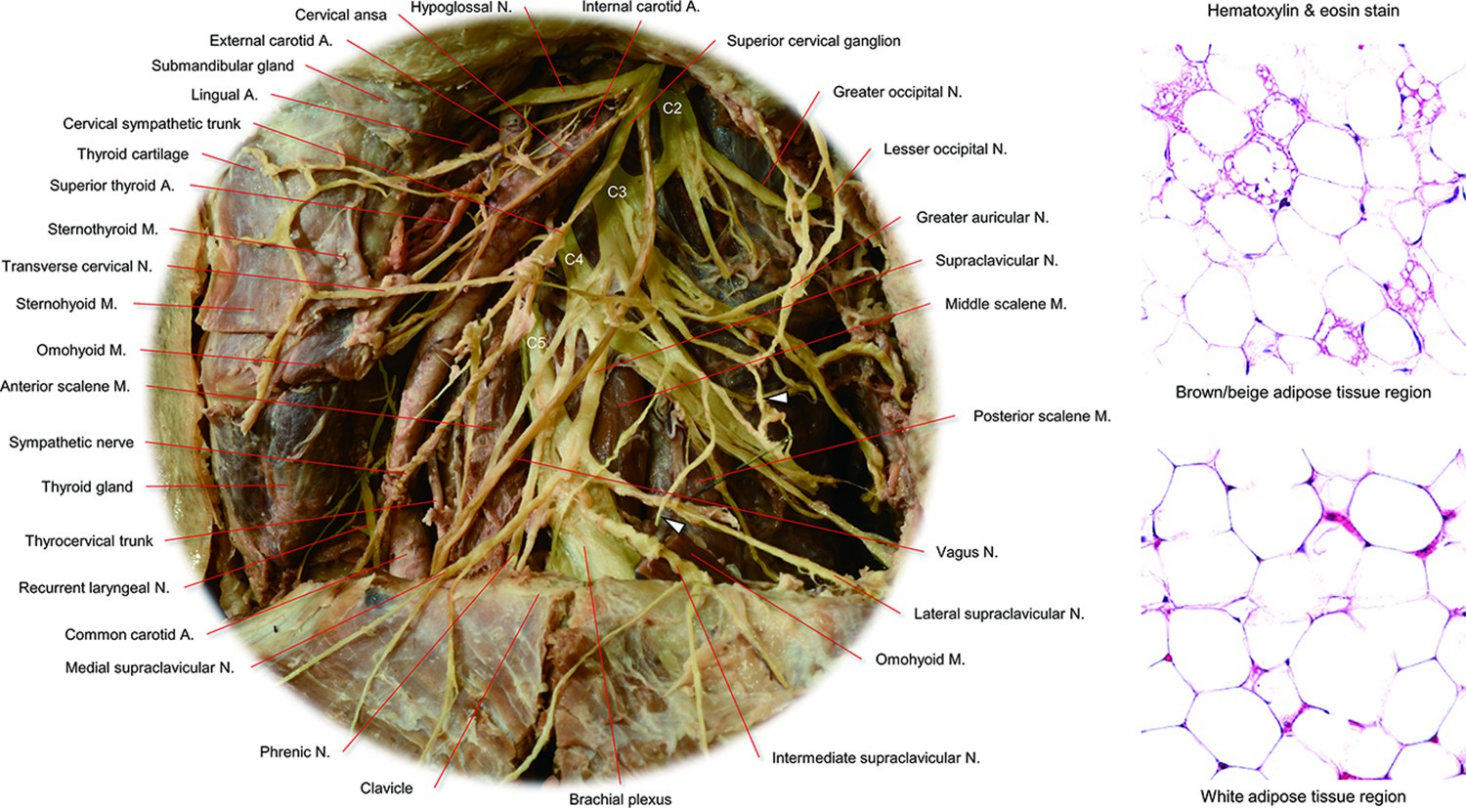
Cervical nerve plexus in the left supraclavicular region. Nerves in the left supraclavicular region are carefully dissected and digitally enhanced with light-yellow (left). Cervical nerve plexus, the sympathetic nerve, and the vagus nerve are shown. Dissection focusing on the innervation of the supraclavicular fat pad is challenging because these nerves can only be visualized after meticulous and thorough removal of the supraclavicular fat pad surrounding these nerves. White arrowheads denote the cut edge of the nerve branch from the 3^rd^ cervical nerve (C3) initially innervating the removed supraclavicular fat pad. Even in the donated elderly bodies, brown adipose tissue (right upper) can be detected histologically in the removed supraclavicular fat pad. Compared to white adipose tissue region (right lower), brown adipose tissue region shows multilocular and smaller adipocytes. A, artery; M, muscle; N, nerve.

**Fig 2:**
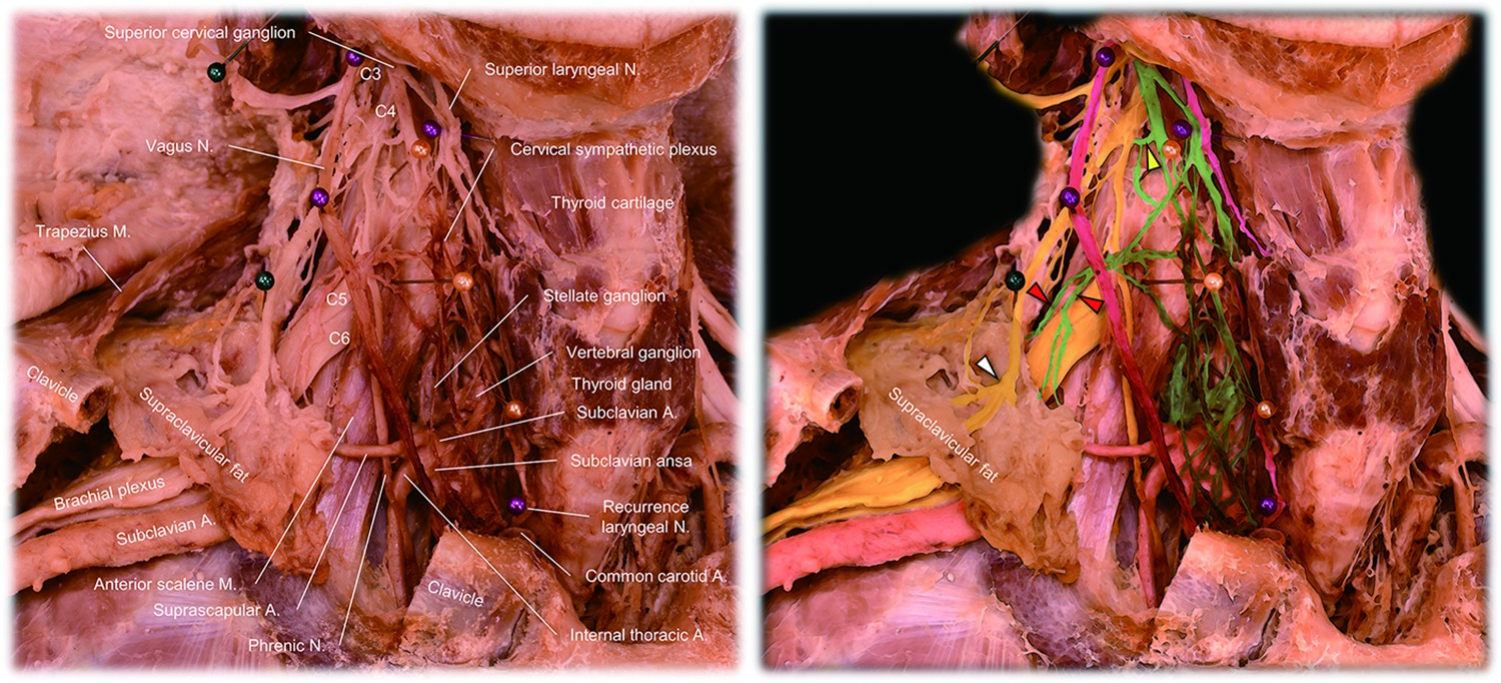
Multiple nerves innervating the right supraclavicular fat pad. Right supraclavicular fat pad is preserved en bloc as far as possible with its innervation (left). The middle part of the right clavicle is removed for a better field of view. Multiple nerve branches innervate this region, and the right suprascapular artery is running in this region. The cervical nerves, vagus nerve and its branches, cervical sympathetic plexus, and arteries are digitally enhanced in yellow, purple, green, and pink, respectively. Red arrowheads denote direct sympathetic nerve branches originating from the cervical sympathetic plexus, innervating the supraclavicular fat pad. Yellow arrowhead shows the communicating ramus from the cervical sympathetic nerves to the proximal segment of the 4^th^ cervical nerve (C4). White arrowhead indicates the nerve branch from the C4 innervating the supraclavicular fat pad. A, artery; M, muscle; N, nerve.

**Fig 3:**
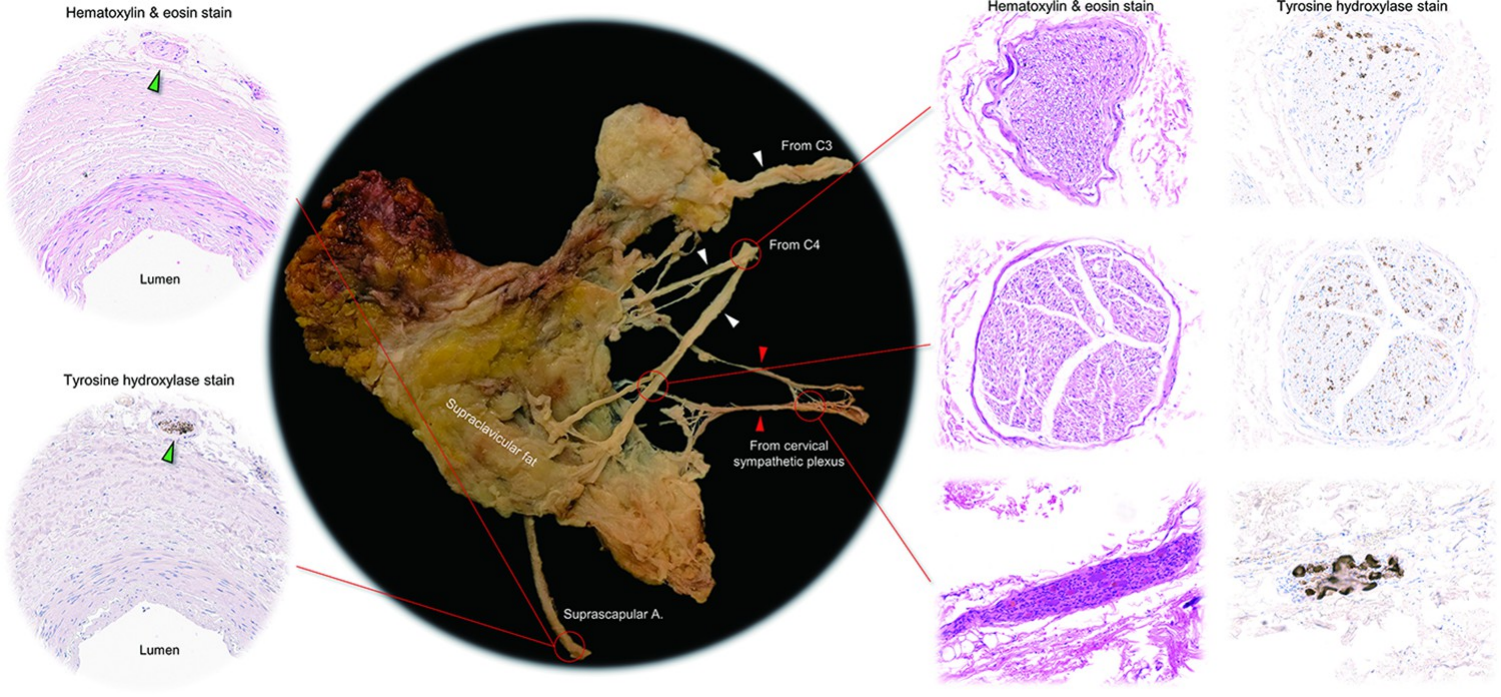
Histology of the multiple nerves innervating the right supraclavicular fat pad. The supraclavicular fat pad in Fig 2 was removed en bloc with its innervating nerves (left). White arrowheads denote the nerve fibers from the 3^rd^ (C3) and 4^th^ (C4) cervical nerves innervating the supraclavicular fat pad. Red arrowheads indicate the thin nerve fibers originating from the cervical sympathetic plexus, which directly innervate the supraclavicular fat pad. The middle and right panels show the hematoxylin & eosin and tyrosine hydroxylase stains of these nerves, respectively. Upper and middle panels show middle and distal part of the C4, respectively. Tyrosine hydroxylase stain shows scattered pattern of sympathetic nerve fascicles. Bottom panels show the nerve from the cervical sympathetic plexus, showing a ganglion involved within the nerve. The suprascapular artery is the main artery running through the supraclavicular fat pad. Histology of the suprascapular artery confirms the sympathetic nerve fibers running along this artery (green arrowheads). A, artery.

The cadaveric donors’ background characteristics are shown in Table 1. Fig 1 shows the complexity of the cervical nerve plexus in the left supraclavicular region. When all the supraclavicular fat pad was removed, the dense and multilayered cervical nerve plexus could be observed with the sectioned edge of the nerve branches terminating in the supraventricular fat pad. The supraclavicular nerves and the transverse cervical nerve were found in the subcutaneous layer, the vagus nerve, sympathetic plexus, and phrenic nerve were found in the middle layer around the common carotid artery, internal jugular vein anterior to the anterior scalene muscle. Proximal segments of the 2^nd^ to 8^th^ cervical nerves were located at the deepest location, originating from the lateral margin of the longus capitis/colli muscle and running on the middle and posterior scalene muscles. While BAT activity decreases with age [20], in the elderly donors of this study, small clusters of BAT could be confirmed in the supraclavicular fat pad samples. In all six cervical dissections, nerve branches from the 3^rd^ and 4^th^ cervical nerves terminating in the supraclavicular fat pad were confirmed. In four out of six supraclavicular fat pad samples, direct sympathetic nerve branches originating from the cervical sympathetic plexus were detected. Fig 2 demonstrates the representative dissection images showing the multiple nerves innervating the supraclavicular fat pad. Nerve branches from the 3^rd^ and 4^th^ cervical nerves and cervical sympathetic plexus were detected. The histology of those nerves confirmed the presence of sympathetic nerve fibers and ganglia (Fig 3). The sympathetic fibers involved in the 3^rd^ and 4^th^ cervical nerves were probably derived from the communication rami between the cervical sympathetic ganglionic plexus and the proximal segment of those cervical nerves (Fig 2) [21, 22]. The suprascapular artery was the main artery running through the supraclavicular fat pad. Histology also confirmed the sympathetic nerve fibers running along this artery (Fig 3).

### Histological evaluation of the proximal 4^th^ cervical nerve

**Fig 4:**
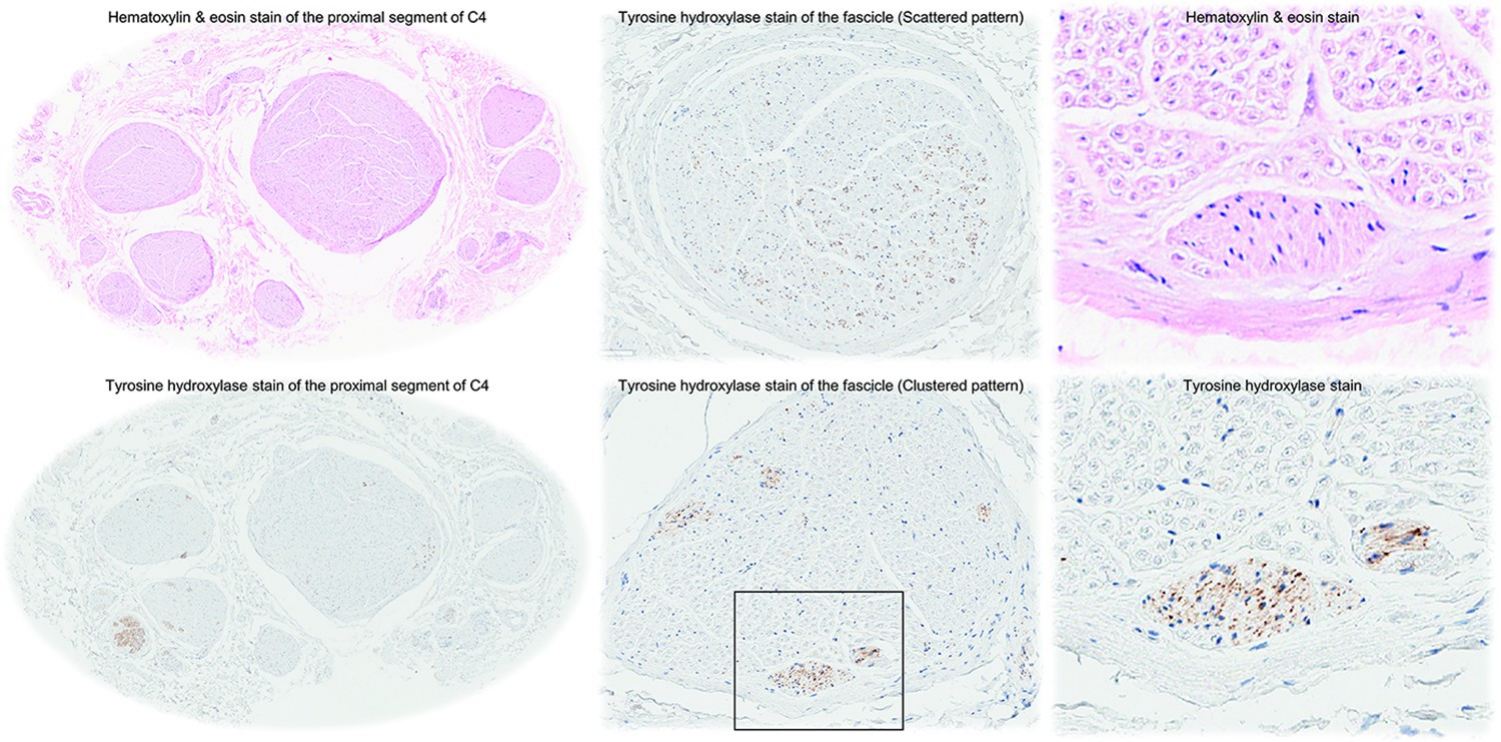
Histology of the proximal segment of the 4^th^ cervical nerve. The proximal segment of the 4^th^ cervical nerve (C4) just lateral to the longus capitis muscle is cross-sectioned and stained with hematoxylin & eosin and for tyrosine hydroxylase. The nerve involves multiple fascicles (left). Middle panels show representative single fascicles showing scattered (upper) and clustered (lower) patterns. The right panels show magnified images of the black-rimmed box in the middle lower panel, showing the cluster of fibers stained positive for tyrosine hydroxylase located at the rim of the fascicle adjacent to the perineurium. Although specific myelin stain was not carried out, these fibers are likely to be unmyelinated postganglionic nerve fibers from the morphological observation with hematoxylin & eosin stain.

The donors’ background characteristics are shown in Table 2. Cross-sectional images of the proximal segments of the 4^th^ cervical nerves were stained with hematoxylin & eosin and tyrosine hydroxylase. This revealed multiple fascicles (Fig 4) in each nerve; on average 10 fascicles. Cross-sectional size of the nerves was 4.3 mm × 2.7 mm on average. Every fascicle included tyrosine hydroxylase positive nerve fibers, showing scattered or clustered patterns of distribution. The scattered pattern was more predominant than the clustered pattern (80% vs. 20% on average). Although a specific myelin stain was not performed, when compared with hematoxylin & eosin stain, the tyrosine hydroxylase positive fibers were likely to be unmyelinated postganglionic fibers, and this was in contrast to the predominant myelinated fibers (Fig 4). These sympathetic nerve fibers occupied only 2.5% of the nerve cross sectional area on average.

## Discussion

With emerging interest in BAT [23], multiple questions have been asked about its distribution [24, 25], the reliability of methods to measure its volume and activity [2, 5, 26, 27], the role and quantification of beige adipose tissue [28], its relationship with obesity [29], its endocrine function [3], and its innervation [19]. Clarifying these issues is fundamental in reaching the final clinical goal which would be selective regulation of its activity either using chemical, mechanical, electrical, or thermal therapeutic approaches to control metabolic disorders [30]. With regard to innervation of BAT, considering the decades of studies related to BAT, it is surprising that the relevant anatomy had not been fully described in humans until the study by Sievers et al. This may be, in part, due to technical difficulties because techniques used in rodent models such as retrograde viral trans-neuronal tract tracer (pseudorabies virus) and immunolabeling-enabled 3-dimensional imaging of solvent-cleared organs [31, 32] cannot be performed in humans, and visualization of the nerves innervating supraclavicular fat pad in cadaveric tissue requires meticulous dissection to remove the supraclavicular fat pad and relevant nerves (Fig 1). Our goal, in this project, was to better define pathways by which sympathetic activation of BAT, which is thought to be the most significant stimulus to BAT activation [31] is likely to take place, and then contemplate potential sites for therapeutic peripheral nerve stimulation. We applied a staged layered approach to dissection in order to maintain intactness of the supraclavicular fat deposit. As a result, we found multiple sympathetic pathways innervating the supraclavicular fat pad, including those from the 3^rd^ and 4^th^ cervical nerve [19], and those from the cervical sympathetic plexus. Histological evaluation of the 4^th^ cervical nerve confirmed the existence of sympathetic nerve fibers in every fascicle with either scattered or clustered distribution pattern.

### Potential innervation pathways to the supraclavicular fat pad

**Fig 5:**
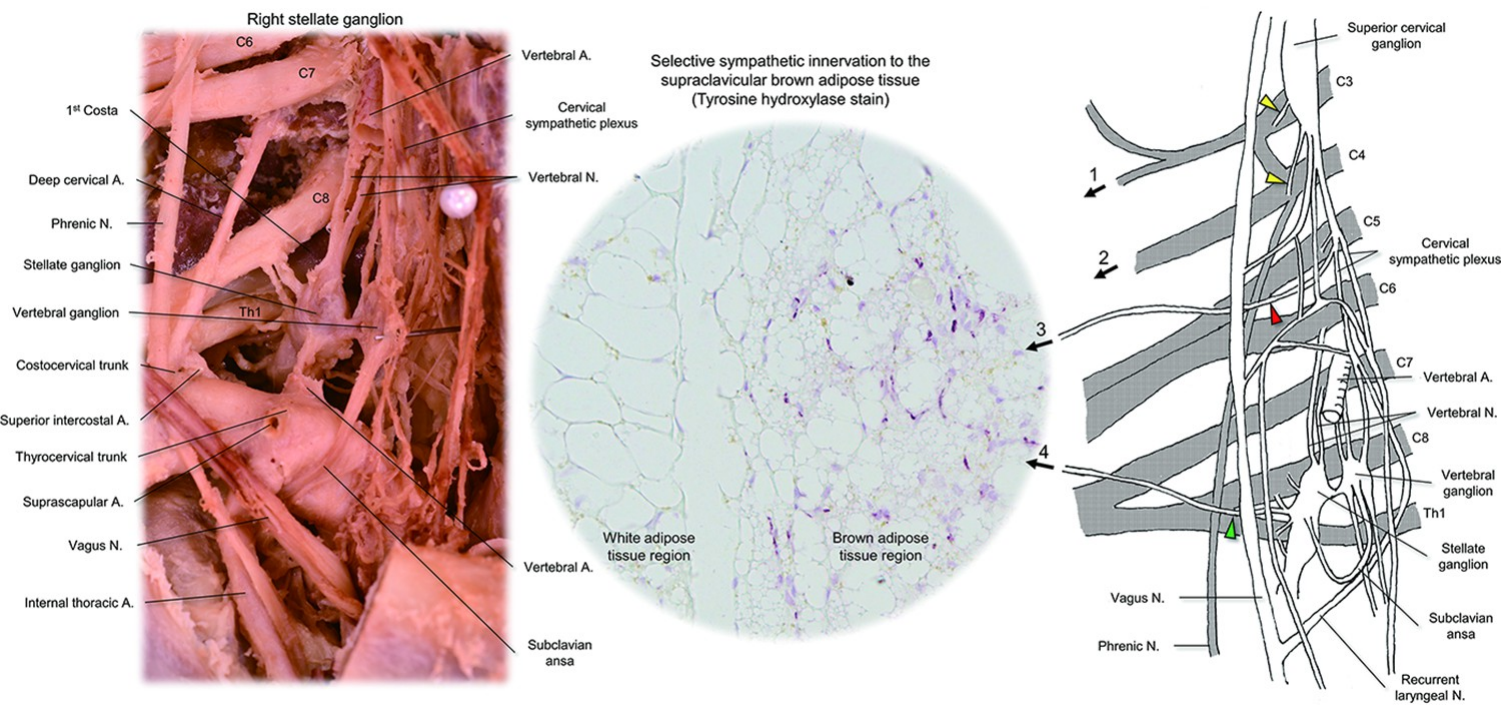
Estimated sympathetic pathways innervating the supraclavicular fat pad. Left panel shows the real dissection of the human right stellate ganglion. Middle panel shows the representative histology of the supraclavicular fat pad obtained from cervical surgery. Tyrosine hydroxylase stain reveals selective sympathetic innervation to the supraclavicular brown adipose tissue. The right panel shows the schematic illustration of potential pathways (1-4) innervating the supraclavicular fat pad. Those involve nerve branches from the 3^rd^ cervical nerve (C3) (route 1), 4^th^ cervical nerve (C4) (route 2), cervical sympathetic plexus (red arrowhead) (route 3), and the stellate ganglion (green arrowhead) along the suprascapular artery (Figs 2 and 3) (route 4). Yellow arrowheads denote communicating rami from the cervical superior cervical ganglion to the proximal segment of the C3 and C4. The direct connection between the sympathetic nerve branch running along with the suprascapular artery and the stellate ganglion itself could not be identified in our dissection. A, artery; N, nerve.

Based on the present findings, an illustration was created to show estimated pathways by which sympathetic nerves innervate the supraclavicular fat pad (Fig 5). At this point, potential sympathetic innervation involves nerve branches from the 3^rd^ cervical nerve (route 1), 4^th^ cervical nerve (route 2), cervical sympathetic plexus (route 3), and the stellate ganglion along the suprascapular artery (route 4). Although it requires further investigation to confirm direct innervation from the stellate ganglion (route 4), we confirmed multiple anatomical innervation pathways originating from the stellate ganglion. The dominance of this ganglion as the central terminus regulating neuronal pathways to human supraclavicular BAT is strongly suggested by the demonstration in vivo that unilateral surgical ablation of this ganglion completely blocks the uptake of 18F-fluorodeoxyglucose positron emission tomography/computed tomography by supraclavicular BAT compared to normal contralateral supraclavicular fat uptake of the tracer in the presence of an intact stellate ganglion [33]. This information might be important when considering a future strategy of targeted neuromodulation of BAT in the supraclavicular fat, perhaps using activation of peripheral nerves rather than the proximally located stellate ganglion. This is potentially important as it would allow a selective response in BAT without increasing concerns for potential side effects from stimulation of other stellate ganglion-related pathways such as to the heart.

Spinal sympathetic outflow is limited to the thoracolumbar outflow with prominent small medullated fibers (under 3 μm in diameter) in the ventral roots, eventually communicating with the thoracic sympathetic chain through the white rami communicans. Those preganglionic (myelinated) fibers either synapse in the sympathetic ganglion at that level, and the postganglionic (unmyelinated) fibers reenter the spinal nerve via gray rami communicans. Otherwise, without synapsing, the preganglionic fibers ascend in the cervical sympathetic chain. After synapsing in the cervical ganglia, the postganglionic fibers join the cervical nerves via the gray rami communicans. Generally, the large superior cervical ganglion gives off gray rami communicans to the 1^st^ to 4^th^ cervical nerves. This concept has been widely confirmed with functional evidence. Thus, it was rather surprising to find a diffusely scattered distribution of sympathetic fibers in the proximal segment of 4^th^ cervical nerve (Table 2). As the source of these sympathetic fibers should be derived from the gray rami communicans of the cervical sympathetic plexus (Figs 2 and 5), we had expected a clustered pattern near the perineurium in the proximal segment near the communication. This observation may reflect the variation of the communication site compared to the sampling segment. How the fibers spread into each fascicle requires further investigation, but the present observation of predominant scattered pattern may demonstrate that these nerve fibers innervate a large expanse supraclavicular fat pad. Similar variable findings have been reported in the distribution pattern of the sympathetic nerve fibers in human cervical and thoracic vagus nerves [34].

Another potential hypothesis for the origin of sympathetic fibers found in the proximal segment of the 4^th^ cervical nerve is described below. Although spinal sympathetic outflow is limited to the thoracolumbar outflow as mentioned above, the cervical ventral roots in humans does have a small amount of small medullated fibers, though they rarely number more than 100 per root [35]. Thus, anatomically, the boundaries of thoracolumbar sympathetic outflow are blurred. Although the significance of this small number of potential preganglionic sympathetic fibers in the cervical ventral roots has not been clear [35], it could be the potential source of the sympathetic outflow to the supraclavicular BAT through the 3^rd^ or 4^th^ cervical nerve after relaying the pre- and postganglionic fibers at the superior cervical ganglion to take a short-cut bypassing the conventional pathway via the stellate ganglion. Histological reevaluation of the cervical ventral roots and functional investigation is necessary to confirm this hypothesis by stimulating the cervical ventral roots and measuring the supraclavicular BAT activity.

Predominant myelinated fibers found in the proximal segment of 4^th^ cervical nerves, axons of which measure 5~10 μm, are likely to be the sensory afferent fibers of supraclavicular region. It has been speculated that substance P and calcitonin gene related peptide sensory nerve fibers innervate BAT to change BAT growth (mass, protein content), mitochondrial content and thermogenic capacity (uncoupling protein-1 content) in rats [36, 37]. Hence, the predominant myelinated fibers may have a regulatory role for human BAT deposits.

### Clinical implications

**Fig 6:**
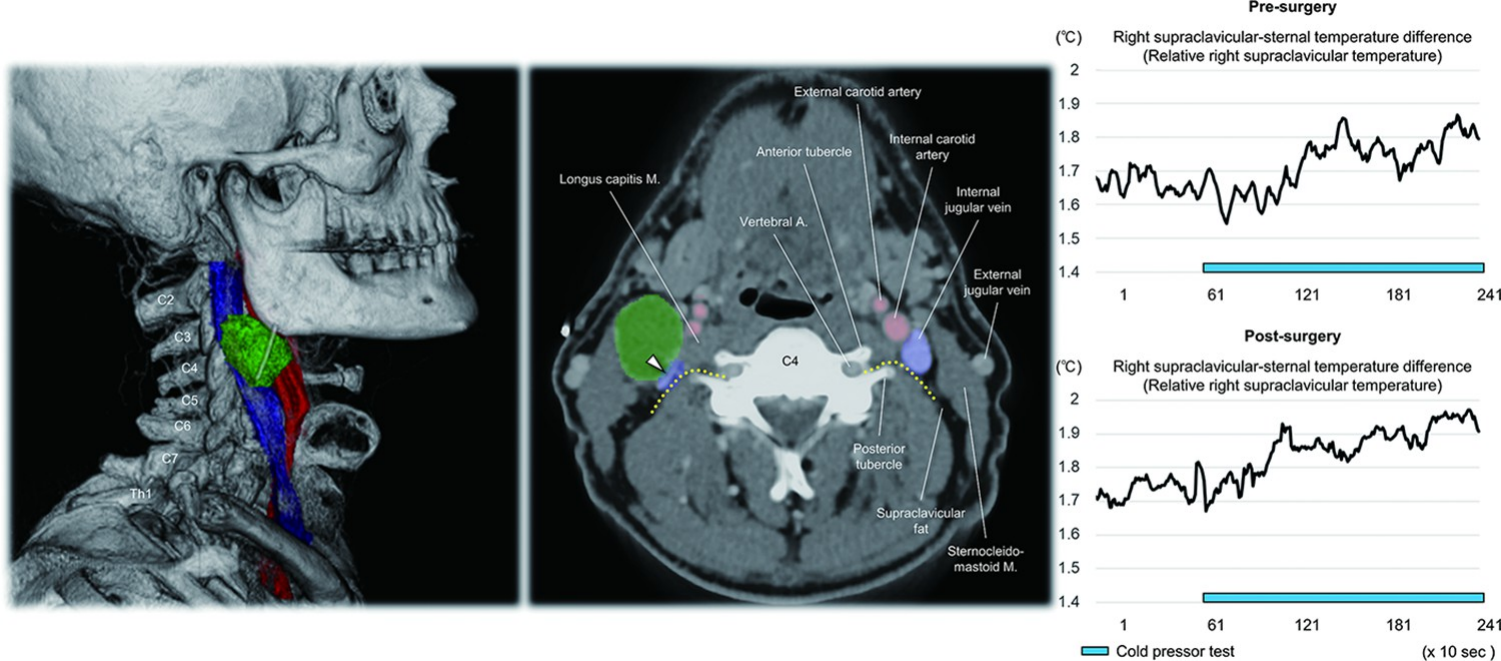
Patient with a cervical tumor and infrared thermography finding. Volume-rendered image of the computed tomography viewed from the right lateral direction (left) shows cervical tumor (green) adjacent to the right internal jugular vein (blue) and carotid artery (red). The location of the tumor is 3^rd^ (C3) to 4^th^ (C4) cervical nerve level. Horizontal section viewed from the inferior direction (middle) confirms compression of the right internal jugular vein (white arrowhead), which is also close to the estimated location of the proximal segment of the C4 (yellow dotted lines) just lateral to the longus capitis muscle. Right panels show the pre- and post-surgical result of the infrared thermography of the right supraclavicular region. The increase in the relative temperature (difference between the sternal and supraclavicular temperatures) in response to the cold pressor (blue bar) increased further after surgery (Δ0.164°C to Δ0.270°C). A, artery; M, muscle.

The present anatomical findings suggest that multiple sympathetic nerve branches extending to the supraclavicular region are involved in BAT innervation (Fig 5). Any pathology involving these nerves can potentially alter the activity of supraclavicular BAT. Fig 6 shows the case of a patient with a right cervical squamous cell carcinoma at the level of 3^rd^ and 4^th^ cervical nerve. The estimated location of the proximal segment of the 4^th^ cervical nerve, emerging between the vertebral artery and posterior tubercle of the transverse process, as well as internal jugular vein, were compressed by the tumor. In a clinical series of patients with cervical tumors, we have evaluated dynamic BAT response to a cold pressor test using infrared thermography [16, 38] in the pre- and post-surgical setting. When focusing on the change in the relative temperature (difference between supraclavicular temperature and sternal temperature), which reflects the activity of BAT [16], we have observed depletion of the thermogenic response to the cold pressor before the surgery. Interestingly, the normal thermogenic response is restored after surgery (Fig 6), suggesting the relief from the mechanical compression (unpublished data). This clinical finding presents two intriguing points we are considering further in our clinical evaluations of BAT:

1. The hierarchy of BAT response to sympathetic innervation, including the relative importance of sympathetic innervation from C3/4 fibers, versus branches from the cervical sympathetic plexus versus branches from the stellate ganglion needs to be considered. The clinical scenario presented suggests that even a focal compression of sympathetic innervation (from the post ganglionic upper cervical routes either through C3/4 or via the cervical plexus directly) causes decrease in BAT thermogenic activity.

2. Considering the key role of the stellate ganglion (Fig 5) as another important source of postganglionic sympathetic nerve fibers using any of routes towards supraclavicular fat (Fig 5), it will be important to confirm the activity of the supraclavicular BAT and metabolic profiles in patients who undergo interventions involving the stellate ganglion, e.g., percutaneous block or stellate ganglionectomy.

### Limitations

While our study included only a limited number of very meticulous dissections, the consistent nature of our findings is evident (Table 1). Lack of evident sympathetic fibers originating from the cervical sympathetic plexus in two regions are likely to be due to technical difficulties in dissecting out such fine fibers. Second, as this study is based on donated cadavers, high age group and concomitant, well known decline in brown adipocytes, did not allow us to systematically characterize BAT in supraclavicular fat pad, as previously reported [39]. However, even in these elderly donors, we could confirm the multiloculated smaller adipocytes compared to larger white adipocytes (Fig 1). Last, as we could not perform myelin specific stain, we could not confirm that thin fibers observed in histological samples were either unmyelinated or thinly-myelinated fibers (Fig 4). However, as they appear to represent postganglionic sympathetic fibers, they are likely to be unmyelinated.

### Conclusions

Human sympathetic nerves use multiple pathways to innervate the supraclavicular BAT. The present findings will serve as an important first step in gaining a fundamental anatomical understanding, which will subsequently allow us to develop clinical therapeutic approaches to activate human BAT in the supraclavicular region.
